# The effects of a probiotic formulation (*Lactobacillus rhamnosus* and *L. helveticus*) on developmental trajectories of emotional learning in stressed infant rats

**DOI:** 10.1038/tp.2016.94

**Published:** 2016-05-31

**Authors:** C S M Cowan, B L Callaghan, R Richardson

**Affiliations:** 1 School of Psychology, The University of New South Wales, Sydney, NSW, Australia; 2 Department of Psychology, Columbia University, New York, NY, USA; 3 Department of Psychiatry, The University of Melbourne, Melbourne, VIC, Australia

## Abstract

Recently, scientific interest in the brain–gut axis has grown dramatically, particularly with respect to the link between gastrointestinal and psychiatric dysfunction. However, the role of gut function in early emotional dysregulation is yet to be examined, despite the prevalence and treatment resistance of early-onset psychiatric disorders. The present studies utilized a developmental rodent model of early-life stress (ELS) to explore this gap. Rats were exposed to maternal separation (MS) on postnatal days 2–14. Throughout MS, dams received either vehicle or a probiotic formulation (previously shown to reduce gastrointestinal dysfunction) in their drinking water. Replicating past research, untreated MS infants exhibited an adult-like profile of long-lasting fear memories and fear relapse following extinction. In contrast, probiotic-exposed MS infants exhibited age-appropriate infantile amnesia and resistance to relapse. These effects were not mediated by changes in pups’ or dams’ anxiety at the time of training, nor by maternal responsiveness. Overall, probiotics acted as an effective and non-invasive treatment to restore normal developmental trajectories of emotion-related behaviors in infant rats exposed to ELS. These results provide promising initial evidence for this novel approach to reduce the risk of mental health problems in vulnerable individuals. Future studies are needed to test this treatment in humans exposed to ELS and to elucidate mechanisms for the observed behavioral changes.

## Introduction

The experience of early-life stress (ELS) is known to increase vulnerability to a range of psychopathologies, including anxiety.^1, 2, 3^ This is particularly concerning given that anxiety disorders emerging during childhood or adolescence tend to be symptomatically more severe and more resistant to treatment compared with adult-onset disorders.^4, 5^ Treatments that target at-risk children, such as those exposed to ELS, could be particularly effective in reducing the prevalence and severity of early-onset disorders. However, the use of traditional psychotropic medications during development is controversial, in part because of a lack of information about long-term effects for the developing central and peripheral nervous systems.^6^ Thus, it is important to identify alternative methods of reducing the risk of psychopathology using treatments that may affect the central nervous system indirectly.

One potential target here is the brain–gut–microbiota axis. This complex network comprises the brain, the gastrointestinal system, and its resident bacteria (the microbiota, consisting of a staggering 10^13^–10^14^ microorganisms).^7^ Accumulating evidence points to a strong bidirectional communication between these structures that is amplified in conditions of stress.^7, 8^ Epidemiological research and animal studies of the maternal separation (MS) model show that ELS causes dysregulation throughout the brain–gut–microbiota axis, rendering exposed individuals susceptible to both gastrointestinal and mental health problems.^9, 10, 11, 12, 13, 14, 15, 16^ Although most studies of MS have focused on outcomes for adult animals, pathophysiological alterations in colonic function and hypothalamic–pituitary–adrenal axis hyperactivity are observable from a young age.^17^ We have also demonstrated early changes in emotional function, with MS infants exhibiting precocious emergence of adult-like fear relapse and extended fear retention.^18, 19^ It has been suggested that such emotional and gastrointestinal changes may be linked, establishing a case for the investigation of enteric treatments for emotional disorders.^20, 21^

Indeed, there is promising preliminary evidence to support the use of gastroenteric interventions to reduce the impact of ELS. For example, probiotics (microorganisms that deliver health benefits to the host via transient colonization of the gastrointestinal tract)^22^ have been shown to reduce depression-like behaviors in adult MS animals.^23^ In addition, administration of a probiotic formulation, Lacidofil, during MS normalizes gut function and corticosterone release in stressed pups.^17^ At this stage, it is unclear what effect probiotic treatment has on the stress hormone levels and gastrointestinal health of unstressed animals. However, it is evident that probiotics restore at least some aspects of normal development and adult behavior following ELS, suggesting that probiotic treatments hold potential clinical value for stress-exposed individuals. Therefore, the aim of the current series of experiments was to establish whether probiotic treatment might also prevent the alterations in emotional development observed following ELS. Specifically, this exciting possibility was explored by examining whether Lacidofil could rescue normal developmental trajectories of fear retention and extinction in infant rats exposed to MS stress.

## Materials and methods

### Subjects

Experimentally naive Sprague–Dawley-derived rats were bred and housed in the School of Psychology at The University of New South Wales. Rats were housed with their dam and littermates (culled to eight pups per litter) and maintained on a 12-h light/dark cycle (lights on at 0700 hours) with food and water available *ad libitum*. The day of birth was designated postnatal day (P)0. Only male pups were used, with the exception of Experiments 3b and 4 where dams were tested. Animals were randomly allocated to groups with no more than one rat from each litter per experimental group. Group sizes for behavioral experiments were determined based on previous experiments conducted with infants and adults in our laboratory, as well as animal availability (see figure captions for exact group sizes). All data collected for this series of experiments have been reported in the present paper. All animals were treated in accordance with *The Australian Code of Practice for the Care and Use of Animals for Scientific Purposes 7th Edition* (2004), and the Animal Care and Ethics Committee at The University of New South Wales approved all procedures.

### Apparatus

In Experiment 1, a set of two identical rectangular chambers (13.5 cm long × 9 cm wide × 9 cm high) was used. The front and rear walls and ceiling were clear Plexiglas, whereas the floor and side walls consisted of stainless steel rods spaced 1 cm apart. The floor was connected to a custom-built constant-current shock generator, and two high-frequency speakers were fitted on either side of the chamber. In Experiment 2, a second set of rectangular chambers (Context B) was also used that was distinct from the chambers described above (Context A) in both size and visual characteristics. The chambers in Context B were larger (30 cm long × 30 cm wide × 23 cm high), and all four walls and the ceiling were clear Plexiglas, with only the floor being constructed of stainless steel rods. Two side walls were covered with vertical black and white stripes (5 cm wide), and two high-frequency speakers were mounted on the ceiling. All experimental chambers were individually housed in sound-attenuating wood cabinets fitted with a white light-emitting diode (LED), an infrared LED and an infrared camera. The infrared LED provided the sole source of illumination in Context A, whereas both white and infrared LEDs were used in Context B. Ventilation fans in each cabinet produced a constant low-level (50 dB) background noise. Chambers were wiped clean with tap water following each experimental session.

The wood elevated-plus maze (EPM) used in Experiment 3 consisted of two open arms (10 cm wide × 50 cm long with a 1-cm raised edge) and two enclosed arms (10 cm wide × 50 cm long with 39-cm high walls) extending from a central platform (10 cm × 10 cm). The entire apparatus was raised 51 cm above the floor and a video camera was mounted above the maze. The EPM was wiped clean before and after experimental sessions with tap water (during testing of infants) or ethanol (during testing of dams).

### Maternal separation

On P2–14, all pups from a given litter were placed together in an incubator, separate from the dam, for 3 h per day, commencing between 0800 and 1200 hours. The ambient temperature was maintained at ~27 °C with a heat pad. Three centimeters of bedding were provided so that pups could behaviorally thermoregulate as needed.

### Probiotic treatment

A commercially available probiotic formulation, Lacidofil, was provided by Lallemand Health Solutions (Montreal, QC, Canada). Lacidofil comprises live *Lactobacillus rhamnosus* strain R0011 and *L. helveticus* strain R0052 in the ratio 95:5. This particular formulation has been used in a number of published studies (for review, see Foster *et al.*)^24^ and was chosen based on previous findings that it normalizes corticosterone levels in MS infants.^17^ Powdered Lacidofil was rehydrated in distilled water at a concentration of 10^9^ colony-forming units per milliliter, a dosage based on previous studies using the same probiotic preparation,^25^ and provided in dams’ drinking water from P2 to P14. Distilled water was provided in the vehicle condition. All drinking solutions were changed every second day to ensure bacteria viability.

SYBR Green-based quantitative polymerase chain reaction (qPCR) was used to confirm the presence of *L. rhamnosus* R0011 in pups’ stomach milk (Experiment 5). Stomach-milk extraction was performed based on the procedure described by Fellows and Rasmussen.^26^ DNA was extracted using a milk bacterial DNA isolation kit (Norgen Biotek, Thorold, ON, Canada). qPCR was conducted using primers described by Gareau *et al.*^17^ and obtained from Thermo Fisher Scientific (Waltham, MA, USA). A melt curve analysis verified the reaction specificity. See Supplementary Methods for further details.

### Behavioral procedures

During conditioning in Experiments 1 and 2, rats were placed in Context A where, after a 2-min adaptation period, six pairings of a white noise conditioned stimulus (CS; 8 dB above background, 10 s duration) and a shock unconditioned stimulus (US; 0.6 mA, 1 s) were presented. The US was administered in the final second of the CS, and the intertrial interval ranged from 85 s to 135 s, with a mean of 110 s. Rats were returned to their home cages 30–60 s after the final CS–US pairing.

In Experiment 2, rats were given extinction training the day after conditioning. Extinction was conducted in Context B and consisted of a 2-min adaptation (baseline) period followed by 30 non-reinforced presentations of the 10-s CS with a 10-s intertrial interval. Rats were returned to their home cages 30–60 s after the final CS presentation. Freezing was scored using a time-sampling procedure, whereby each rat was scored as freezing or not freezing every 3 s during the baseline period and each presentation of the CS. For analysis, data from the 30 extinction trials were collapsed into five blocks of six trials each.

During test for Experiments 1 and 2, levels of freezing were recorded throughout a 1-min adaptation period (baseline) and a 2-min presentation of the CS.

In Experiment 3, the EPM was used as a measure of anxiety. On P17, either the infants or dams were placed in the center of the EPM facing a closed arm. Animals were considered to have entered an arm when the two front paws and the head were on the arm. The latency to enter an open arm, number of entries into open and closed arms, and time spent in open and closed arms were recorded for 5 min, after which animals were returned to the home cage.

For Experiment 4, maternal behavior was assessed using a pup-retrieval test conducted on P6, when pups are still relatively immobile. Dams were removed from the home cage for 7 min, during which time pups were removed from the nest and placed on the opposite side of the home cage (24.5 cm wide × 37 cm long). On reunion, dams were placed back in the home cage on the opposite side to pups. The time taken to retrieve pups to the nest was observed and recorded from an adjacent room via a video camera positioned above the cage. Dams that did not retrieve all pups to the nest within a 10-min period were scored at the maximum value (600 s); the same statistical results were obtained if these litters were excluded from the final analysis.

### Exclusions and statistics

As high levels of baseline freezing make it difficult to detect CS-elicited freezing, any rat that exhibited >60% baseline freezing was excluded from the final analysis. This resulted in two exclusions (one rat from Group Vehicle—7 days in Experiment 1 and one rat from Group Probiotic—Different in Experiment 2). In addition, one rat was excluded from Group Probiotic—Different in Experiment 2 as it was a statistical outlier (3.7 s.d. from the mean). Due to significant differences in baseline freezing between groups (Supplementary Table S1), data are presented and analyzed as difference scores (percent freezing during the CS minus percent freezing during baseline). The same pattern of results was obtained if the data were analyzed by analysis of variance (ANOVA) with the raw data (ignoring baseline scores) or by analysis of covariance with baseline freezing as a covariate. Using Levene’s test of equality of variances, variance was found to be similar for all reported comparisons (see also s.e.m. values in relevant figures). Two-tailed tests were used throughout, with values of *P*<0.05 considered statistically significant. Where the assumption of sphericity was violated, the Greenhouse Geisser procedure was used but nominal df are reported. Where the assumption of normality was violated, non-parametric tests were conducted; in all cases the same statistical results were obtained, so the ANOVA statistics are presented for consistency. A random sample (30%) of the test data was cross-scored by a second observer unaware of subjects’ experimental condition, with high inter-rater reliability across all experiments (*r*s=0.97–1.00).

## Results

### Dam fluid intake

Fluid consumption was monitored daily throughout treatment (Supplementary Figure S2). Intake increased with pups’ age for both treatment types. Probiotic-treated dams consumed more than vehicle-treated dams, although only in the first few days of treatment (see Supplementary Results for statistical analysis).

### Animal weights

Dams and pups were weighed daily throughout MS (Supplementary Figure S3). Pups gained weight across development, whereas dams lost weight (see Supplementary Results for analysis). There were no significant differences in weight between treatment groups, indicating that caloric intake was similar despite the initial difference in fluid intake. In other words, any potential change in nutritional intake in the probiotic-exposed animals was not reflected in a macro measure of energy intake (that is, weight gain).

### Experiment 1: Effect of probiotics on infantile amnesia in MS infants

Previous work from our laboratory has shown that ELS results in a precocious transition to an adult-like memory system.^19, 27^ Infantile amnesia is a well-established phenomenon, whereby standard-reared (SR) infants exhibit rapid forgetting of learned fear.^28, 29^ However, MS infants retain and express fearful associations for extended periods of time.^19^ In Experiment 1, we tested the hypothesis that a probiotic treatment shown to reduce the hormonal and gastrointestinal impact of MS^17^ would also reverse the impact of MS on infant fear retention.

A 2 × 2 between-subjects design was employed, with the factors referring to treatment (probiotic or vehicle) and test interval (1 or 7 days). MS infants were conditioned on P17 and tested either 1 or 7 days later.

**Figure 1:**
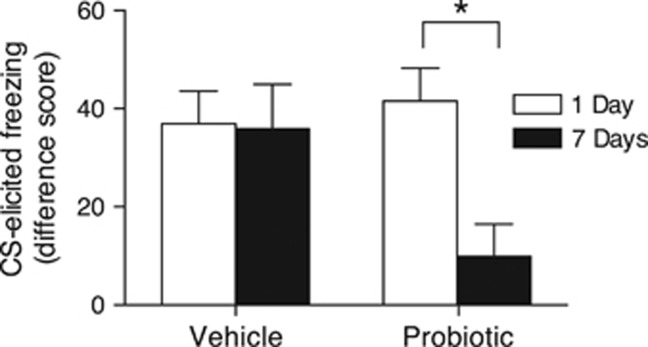
Mean (±s.e.m.) CS-elicited freezing at the retention test for vehicle- and probiotic-exposed maternally separated (MS) infant rats. Vehicle-exposed MS rats exhibited excellent retention at both 1 day (*n*=14) and 7 days (*n*=13). Probiotic-exposed MS rats exhibited good retention at the 1-day interval (*n*=13), but age-appropriate forgetting (that is, infantile amnesia) after 7 days (*n*=10). *P<0.05. CS, conditioned stimulus.

Test results are presented in Figure 1. The effect of treatment was nonsignificant (*F*_1,46_=2.23, *P*=0.142), but there was a significant effect of test interval (*F*_1,46_=5.18, *P*=0.028) and a significant Treatment × Test Interval interaction (*F*_1,46_=4.55, *P*=0.038). Follow-up comparisons revealed that probiotic-exposed rats exhibited significantly less freezing (that is, forgetting) at the 7-day test compared with the 1-day test (*t*_21_=3.46 (95% confidence of interval (CI), 12.64–50.69), *P*=0.002). However, the effect of test interval was nonsignificant for vehicle-exposed MS rats (*t*_25_=0.10 (95% CI, −20.97 to 23.04), *P*=0.924).

The results of Experiment 1 replicate our previous finding that MS infants retain fear memories across extended intervals.^19^ More importantly, treating dams with probiotics restored age-appropriate infantile amnesia in MS pups. This result cannot be attributed to a reduction in initial learning as both groups demonstrated equivalent levels of freezing to the CS after 1 day. Rather, probiotic treatment returned the stressed infants to the expected phenotype of forgetting observed in unstressed infant rats.

### Experiment 2: Effect of probiotics on fear relapse in MS infants

In Experiment 1 we demonstrated that probiotic treatment prevents the precocious transition to adult-like fear retention in stressed infants. ELS also accelerates the transition from a relapse-resistant extinction phenotype to an adult-like phenotype of fear relapse. That is, a number of reports demonstrate that SR infant rodents do not exhibit renewal, reinstatement or spontaneous recovery of extinguished fear.^30^ However, infants exposed to ELS exhibit relapse (at least renewal and reinstatement) following extinction.^18, 27^ Experiment 2 was designed to test whether probiotic treatment would prevent the precocious emergence of relapse-prone extinction in MS infant rats (just as it prevented the precocious transition to adult-like fear retention in Experiment 1).

The design was a 2 × 2 factorial, with the factors referring to treatment and test context. P17 rats were conditioned on Day 1, extinguished on Day 2 and tested on Day 3 in either the same context as extinction or a different context.

**Figure 2:**
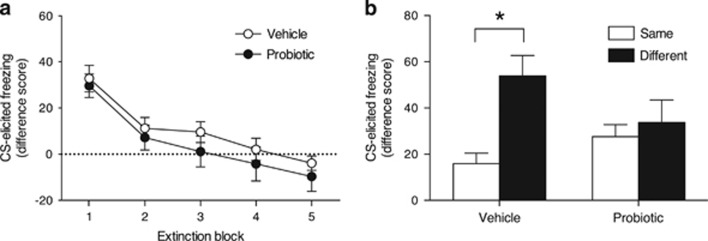
Mean (±s.e.m.) CS-elicited freezing during (**a**) extinction and (**b**) test for vehicle- and probiotic-exposed maternally separated (MS) infant rats. Only vehicle-exposed MS rats demonstrated the renewal effect, that is, higher freezing in the different context (*n*=11) compared with the same context (*n*=11). Probiotic-exposed MS rats exhibited low freezing in both the same context (*n*=15) and the different context (*n*=14). *P<0.05.

*Extinction.* The effects of subsequent test context and related interactions were nonsignificant (largest *F*_1,47_=1.60, *P*=0.212); therefore, extinction data were collapsed across test context. As expected, freezing decreased across extinction blocks (*F*_4,188_=29.11, *P*<0.001; Figure 2a). The effect of treatment and related interactions were nonsignificant (*F*s<1). In other words, treatment did not affect levels of conditioned fear or the rate of extinction.

*Test.* Whereas the effect of treatment was nonsignificant (*F*<1), there was a significant effect of test context (*F*_1,47_=9.30, *P*=0.004) and a significant Treatment × Context interaction (*F*_1,47_=4.82, *P*=0.033; Figure 2b). Follow-up comparisons revealed that vehicle-exposed MS rats exhibited significantly higher levels of freezing in the different context than in the same context (that is, renewal; *t*_20_=4.03 (95% CI, 18.35–57.70), *P*=0.001). However, the effect of context was nonsignificant for probiotic-exposed MS rats (*t*_27_=0.60 (95% CI, −27.50 to 15.12), *P*=0.556).

In Experiment 2, vehicle-exposed MS infants demonstrated context-mediated renewal of extinguished fear, replicating previous findings.^18^ In contrast, probiotic-exposed MS infants did not show this renewal effect. That is, probiotics restored the age-appropriate expression of relapse-resistant extinction. This result is consistent with the findings of Experiment 1, indicating that treating dams with probiotics attenuates MS-induced acceleration of both extinction and fear memory development in pups. Together, these results suggest that a non-invasive probiotic treatment can reduce expression of persistent, relapse-prone fear memories in animals exposed to ELS.

### Experiment 3a: Effect of MS and probiotics on infants’ anxiety

In Experiments 1 and 2 we observed that probiotics reversed the effects of MS on two forms of learned fear. Given reports that MS increases anxiety in adult animals,^13, 14^ it is important to examine whether the anxious phenotype emerges early in life as well. Further, some studies have found anxiolytic effects of probiotics in adult rodents,^31, 32^ suggesting that the present treatment might also influence anxiety levels in pups. It is possible that anxiety at the time of training may have influenced the strength of encoding of the fear memory and thereby influenced test performance. Thus, in Experiment 3a, the EPM was used to measure infants’ anxiety at the age animals were trained in the first two experiments (that is, P17). Three groups of animals were tested on the EPM, a widely used and well-validated measure of anxiety in rodents.^33^ Two groups were exposed to MS with either probiotic or vehicle treatment. A group of SR animals was also tested, as we have not previously compared anxiety in SR and MS infants.

**Figure 3:**
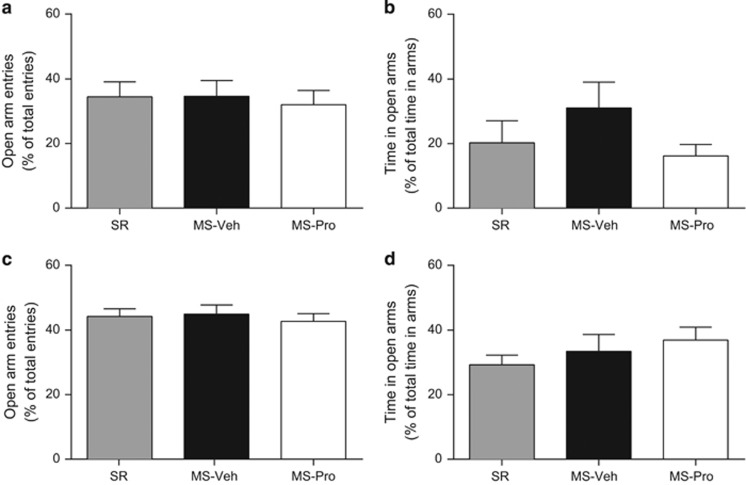
Anxiety-like behavior (mean±s.e.m.) on the elevated-plus maze in (**a, b**) infants and (**c, d**) dams exposed to standard-rearing conditions (SR, pups: *n*=10, dams: *n*=9), maternal separation (MS) with vehicle treatment (MS-Veh, pups: *n*=12, dams: *n*=8), or MS with probiotic treatment (MS-Pro, pups: *n*=12, dams: *n*=8). There were no significant effects of condition on percent entries into open arms or percent time in open arms.

One-way ANOVAs revealed that the effect of rearing condition was nonsignificant for all behavioral measures (largest *F*_2,31_=1.54, *P*=0.229; Figure 3a, b and Supplementary Figure 4a, b). In other words, neither MS nor probiotics altered pups’ anxiety-like behavior or locomotor activity (as measured by total number of arm entries^34^ on the EPM. This would appear inconsistent with previous findings that MS increases anxiety^13, 14^ (but see also Millstein and Holmes^35^ and Eklund and Arborelius^36^) and probiotic treatment decreases anxiety.^31, 32^ However, it is of note that these studies were all conducted in adulthood, whereas in the present study we tested infants. Our finding that MS did not increase anxiety (at least as measured by the EPM) is consistent with the finding that baseline levels of freezing (which can reflect generalized anxiety) are not altered in developing MS animals.^18, 19, 37^ The lack of a probiotic effect in these animals is therefore less surprising in light of previous work showing that some probiotic strains specifically alter mood or behavior only in individuals that are symptomatic.^23, 38, 39^ Overall, the current data suggest that MS-induced differences in retention and extinction of learned fear precede any changes in diffuse levels of anxiety.

### Experiment 3b: Effect of MS and probiotics on dams’ anxiety

Although MS and probiotic treatment did not affect pups’ anxiety in Experiment 3a, it is possible that the effects observed in Experiments 1 and 2 might be mediated by changes in maternal behavior. This hypothesis is supported by research showing that providing dams with a foster litter during MS reduces the effects of separation on offspring hypothalamic–pituitary–adrenal axis development.^40^ Further, it has been shown that MS dams exhibit increased levels of anxiety and depression-like behavior following weaning of pups.^41, 42^ However, to our knowledge, there have been no studies examining changes in maternal anxiety as a result of MS or probiotics while the pups are still under the dam’s care. Thus, Experiment 3b was designed to test whether MS or probiotic treatment alters anxiety levels in active mothers. Similar to Experiment 3a, there were three groups that differed in terms of the rearing conditions they experienced (SR, MS+vehicle and MS+probiotics). SR animals were included as we have not previously compared MS and SR dams on tests of anxiety. Following 2 days of handling, dams were tested on the EPM when pups reached P17.

One-way ANOVAs revealed that the effect of rearing condition was nonsignificant for all measures (*F*s<1; Figure 3c, d, Supplementary Figure 4c, d). That is, MS, with or without probiotic treatment, did not alter dams’ anxiety-like behavior or locomotor activity on the EPM when their pups were 17 days of age (the age at which the pups were trained in Experiments 1 and 2). Whereas there are previous reports of increased anxiety-like behavior in MS dams,^42, 43^ the timing of testing differed vastly between these studies and the current experiment, with all previous work focusing on the period after offspring had been weaned. This would suggest that the effects of MS on maternal anxiety become apparent only after a delay, indicating that developmental alterations in infant fear retention and inhibition are not mediated by changes in maternal anxiety at the time of infants’ training.

### Experiment 4: Effect of probiotics on dams’ maternal behavior

In Experiment 3, we found that alterations in infant fear learning were not mediated by changes in either pup or dam anxiety. An alternate possible explanation for the observed probiotic effect on infant behavior in Experiments 1 and 2 is that there may be changes in mother–infant interactions. For example, there is abundant evidence that naturally occurring variations in maternal behavior can alter long-term neuroendocrine and behavioral outcomes for pups.^44^ Thus, in Experiment 4 we examined pup-retrieval, a species-typical maternal response that is regulated by the hormone oxytocin.^45^

**Figure 4:**
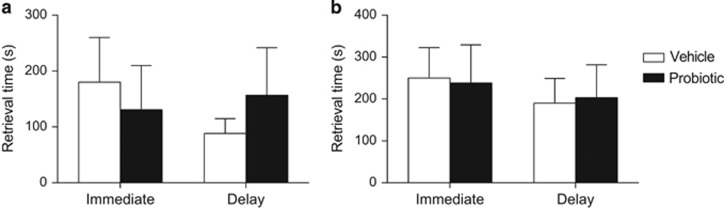
Mean (±s.e.m.) time for vehicle-treated (*n*=9) and probiotic-treated (*n*=7) maternally separated dams to retrieve the (**a**) first pup and (**b**) last pup to the nest. There were no significant effects of treatment on retrieval time, regardless of whether the test was conducted immediately after maternal separation, or at a delay (after a 3-h period of being united).

As shown in Figure 4, probiotic treatment did not alter maternal responsiveness on the pup-retrieval test. This was the case at both test time points (that is, at reunion after 3 h of MS and at a delay, following a 3-h period of being united), with no significant effects or interactions of treatment or test time on the latency to retrieve the first or last pup (largest *F*_1,14_=1.18, *P*=0.296). In other words, the effects of probiotic observed in Experiments 1 and 2 cannot be explained by alterations in maternal care, at least as assessed by pup-retrieval.

### Experiment 5: Detection of probiotics in pups’ stomachs

**Figure 5:**
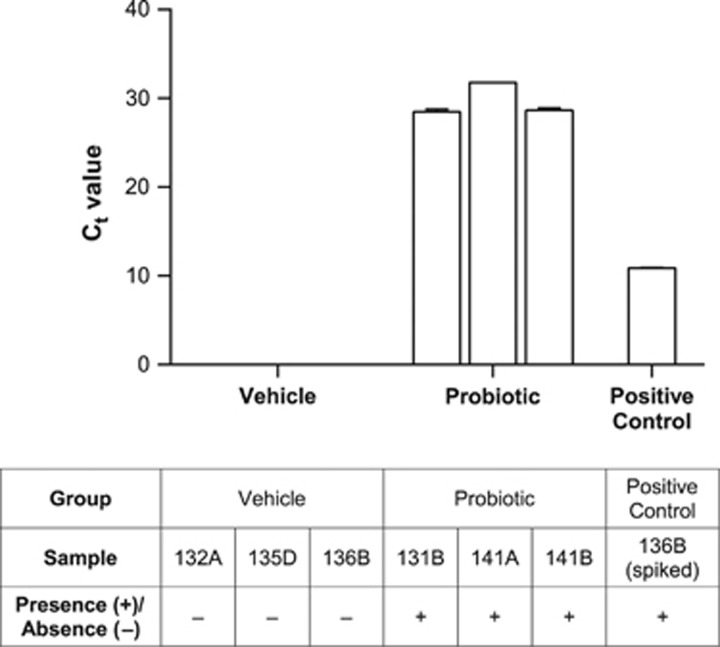
Mean (±s.d.) threshold cycle (C_t_ values) for amplification of *L. rhamnosus* strain R0011 from stomach milk samples of vehicle-exposed (*n*=3) and probiotic-exposed (*n*=3) maternally separated pups at P7, and a positive control prepared from a vehicle-exposed sample spiked with probiotic solution. C_t_ values for all samples in the vehicle-exposed group were undetermined (includes C_t_ values >33 or undetermined by the software).

To assess for the transmission of the probiotic bacteria to pups, stomach contents were extracted on P7 and analyzed using qPCR for the presence of the predominant strain in the probiotic treatment (*L. rhamnosus* R0011). This analysis indicated that *L. rhamnosus* R0011 was present at detectable levels in the positive control as well as in stomach milk samples obtained from probiotic-exposed MS pups (Figure 5). However, no amplification was observed in stomach milk samples obtained from vehicle-exposed MS pups. Thus, it is clear that treatment of nursing dams resulted in transfer of the probiotic to pups, and this was specific to probiotic-exposed animals.

## Discussion

In the present series of experiments we investigated the effects of a probiotic formulation, Lacidofil, on infant and maternal behavior in the MS model of ELS. Replicating previous results from our laboratory,^18, 19^ untreated MS infant rats exhibited adult-like extended fear retention and relapse-prone extinction. However, the MS offspring of probiotic-treated dams exhibited age-appropriate infantile amnesia and relapse-resistant extinction (Experiments 1 and 2, respectively). The reversal of stress-induced accelerated maturation in MS infants was not mediated by changes in pups’ or dams’ levels of anxiety on the EPM (Experiment 3), nor by changes in maternal responsiveness on the pup-retrieval test (Experiment 4). The results of Experiment 5 demonstrated that the probiotic was present in the stomach milk of only the infants from the treated mothers.

Within the growing body of evidence for the importance of brain–gut–microbiota interactions,^46, 47, 48, 49, 50^ the present study has exciting clinical implications for the treatment of individuals exposed to ELS. There is mounting evidence that probiotic treatments can normalize stress-induced changes throughout the brain–gut–microbiota axis. For example, probiotics reduce depressive behaviors in adult MS rats and normalize gastrointestinal and hormonal effects of both ELS and adult stress.^17, 23, 51, 52^ However, to our knowledge this is the first demonstration that probiotics can affect the behavior of young animals exposed to stress. Specifically, our results show that treating nursing dams with Lacidofil prevents accelerated emotional development in MS infants. Whereas maturation and enhanced learning are ordinarily considered advantageous, it is likely that the specific timing of development is functionally important. For example, studies of perceptual systems demonstrate that accelerated development of one sensory system interferes with normal development of other species-typical behaviors.^53, 54^ Thus, by restoring the normal trajectory of fear retention and extinction to levels expected of standard-reared animals, probiotics may act protectively to prevent disruptions to as-yet unidentified systems. Furthermore, preventing inappropriate expression of fear during infancy may have flow-on effects for inappropriate fear expression, or anxiety, in adulthood. Indeed, the importance of early childhood fearful experiences and responding styles is emphasized in many theories of the etiology of psychopathology.^55, 56^

It will be necessary to test the translational value of the present findings through clinical trials. Currently, research examining the effects of probiotics on emotion regulation in humans is sparse but promising. Two studies in healthy populations have demonstrated enhanced mood and lower anxiety as a result of probiotic treatment,^32, 39^ with an additional study demonstrating that probiotics reduced anxiety in a sample of chronic fatigue patients.^57^ Probiotics have also been shown to reduce urinary levels of the stress hormone cortisol^32^ and to alter functional activity of emotion-related brain regions in a sample of healthy females.^58^ Most of these studies examined nonclinical populations and all examined adults, but our results suggest the need to test probiotic effects in those exposed to ELS. For potential future trials, the non-invasive nature of the present treatment is perhaps its most valuable property as it is likely to encourage greater participation and treatment adherence. Furthermore, the specific probiotic formulation we used, Lacidofil, is generally well-tolerated, with no toxicity reported in animal studies and no serious adverse events reported in commercial applications.^24^ Lacidofil has also been used in multiple studies of pediatric populations with various forms of gastrointestinal dysfunction,^24^ paving the way for future clinical trials with individuals exposed to ELS. Clinical testing in this area is imperative, given the highly specific nature of probiotics; different strains are known to have different effects and mechanisms of action^22, 59^ and the action of individual strains may vary depending on context. For example, another probiotic formulation was found to be beneficial for stressed animals but resulted in MS-like changes when administered in the absence of stress.^60^ A limitation of the present study is that the effects of Lacidofil on standard-reared animals was not investigated, which will be an important area for future studies.

Although the emerging evidence, including the present study, suggests that probiotics may be useful in treating stressed individuals, the mechanisms remain unclear. There are a number of potential pathways for the observed effects. In the present case, treatment was administered to pups indirectly via the dam, suggesting that the mother may mediate the changes in pups’ behavior, a hypothesis supported by previous work on the importance of the maternal experience of MS for infant outcomes.^40^ However, we did not observe differences in dams’ levels of anxiety or maternal responsiveness during pups’ early life. Although this does not exclude the possibility that some other aspect of maternal behavior might be altered, the fact that the probiotic was passed on to pups via dams’ breast milk supports a more direct mode of action.

Given the complex nature of the brain–gut–microbiota interaction, it is likely that probiotic colonization would result in multiple changes that may have contributed to the observed effects on pups’ emotional development. First, modulation of immune function is an important intermediary in the communication between the brain and the gut. The sheer size of the gut–environment interface (the body’s largest immune interface) necessitates a complex, regionally diverse immune system that is dependent on the commensal bacteria to develop and maintain immune homeostasis.^61, 62^ The specific probiotic formulation used here has been shown to modulate immune function via downregulation of pro-inflammatory cytokines and restoration of gut barrier function.^24^ This is particularly relevant as MS animals exhibit a pro-inflammatory profile in keeping with the inflammatory hypothesis of depression.^63, 64^ A second possibility is that probiotics serve to produce (or stimulate production of) neuroactive compounds that may influence neural signaling via the enteric nervous system or vagal afferents.^65, 66^ As one example, the serotonergic system has a key role in emotion regulation and is known to be disrupted by ELS.^67, 68^ Interestingly, commensal bacteria and probiotic species have been shown to influence tryptophan metabolism, leading to altered serotonin levels in the plasma, as well as in cortical and subcortical regions of the brain.^66, 69, 70^ Finally, hypothalamic–pituitary–adrenal axis regulation is another important candidate mechanism for the observed effects, particularly in light of our prior research implicating corticosterone in the accelerated development of MS animals^19, 71^ and evidence that Lacidofil normalizes corticosterone levels in MS pups.^17^ Although it will be important for future research to determine the exact mechanism(s) involved, it is clear that, regardless of the pathway, the probiotic formulation acted as a potent treatment to reverse the effects of ELS on infants’ learned emotion-related behaviors in an effective and non-invasive manner.

In conclusion, our data show that probiotic treatment reverses the effects of MS on learned fear responses during infancy, rescuing the normal developmental trajectory in stressed infants. Alongside evidence that probiotics reduce the hormonal response to MS,^17^ these results indicate that further investigation is warranted to explore the clinical implications of these findings for the treatment of individuals exposed to ELS. In addition, elucidation of the mechanisms for the observed behavioral changes will be a critical line of investigation for future research. Overall, the results presented here highlight the utility of animal models in examining the developmental effects of probiotics and add to the growing body of research highlighting the importance of the brain–gut–microbiota axis for mental health and emotional development.

## References

[bib1] Bos K, Zeanah CH, Fox NA, Drury SS, McLaughlin KA, Nelson CA. Psychiatric outcomes in young children with a history of institutionalization. Harv Rev Psychiatry 2011; 19: 15–24. 21250893 10.3109/10673229.2011.549773 PMC3445019

[bib2] Buckingham ET, Daniolos P. Longitudinal outcomes for victims of child abuse. Curr Psychiatry Rep 2013; 15: 342. 23307564 10.1007/s11920-012-0342-3

[bib3] Heim C, Nemeroff CB. The impact of early adverse experiences on brain systems involved in the pathophysiology of anxiety and affective disorders. Biol Psychiatry 1999; 46: 1509–1522. 10599479 10.1016/s0006-3223(99)00224-3

[bib4] Newman DL, Moffitt TE, Caspi A, Magdol L, Silva PA, Stanton WR. Psychiatric disorder in a birth cohort of young adults: prevalence, comorbidity, clinical significance, and new case incidence from ages 11 to 21. J Consult Clin Psychol 1996; 64: 552–562. 8698949

[bib5] Lee FS, Heimer H, Giedd JN, Lein ES, Šestan N, Weinberger DR et al. Adolescent mental health - opportunity and obligation: emerging neuroscience offers hope for treatments. Science 2014; 346: 547–549. 25359951 10.1126/science.1260497 PMC5069680

[bib6] Bonati M, Clavenna A. The epidemiology of psychotropic drug use in children and adolescents. Int Rev Psychiatry 2005; 17: 181–188. 16194789 10.1080/09540260500093768

[bib7] Dinan TG, Cryan JF. Regulation of the stress response by the gut microbiota: implications for psychoneuroendocrinology. Psychoneuroendocrinology 2012; 37: 1369–1378. 22483040 10.1016/j.psyneuen.2012.03.007

[bib8] Mayer EA, Knight R, Mazmanian SK, Cryan JF, Tillisch K. Gut microbes and the brain: paradigm shift in neuroscience. J Neurosci 2014; 34: 15490–15496. 25392516 10.1523/JNEUROSCI.3299-14.2014 PMC4228144

[bib9] Chitkara DK, van Tilburg MAL, Blois-Martin N, Whitehead WE. Early life risk factors that contribute to irritable bowel syndrome in adults: a systematic review. Am J Gastroenterol 2008; 103: 765–774. 18177446 10.1111/j.1572-0241.2007.01722.x PMC3856200

[bib10] Kendall-Tackett KA. Physiological correlates of childhood abuse: chronic hyperarousal in PTSD, depression, and irritable bowel syndrome. Child Abuse Negl 2000; 24: 799–810. 10888019 10.1016/s0145-2134(00)00136-8

[bib11] Kessler RC, Davis CG, Kendler KS. Childhood adversity and adult psychiatric disorder in the US National Comorbidity Survey. Psychol Med 1997; 27: 1101–1119. 9300515 10.1017/s0033291797005588

[bib12] Sanchez MM, Ladd CO, Plotsky PM. Early adverse experience as a developmental risk factor for later psychopathology: evidence from rodent and primate models. Dev Psychopathol 2001; 13: 419–449. 11523842 10.1017/s0954579401003029

[bib13] Kalinichev M, Easterling KW, Plotsky PM, Holtzman SG. Long-lasting changes in stress-induced corticosterone response and anxiety-like behaviors as a consequence of neonatal maternal separation in Long–Evans rats. Pharmacol Biochem Behav 2002; 73: 131–140. 12076732 10.1016/s0091-3057(02)00781-5

[bib14] Ladd CO, Huot RL, Thrivikraman KV, Nemeroff CB, Meaney MJ, Plotsky PM. Long-term behavioral and neuroendocrine adaptations to adverse early experience. Prog Brain Res 2000; 122: 81–103. 10737052 10.1016/s0079-6123(08)62132-9

[bib15] Barreau F, Salvador-Cartier C, Houdeau E, Bueno L, Fioramonti J. Long-term alterations of colonic nerve-mast cell interactions induced by neonatal maternal deprivation in rats. Gut 2008; 57: 582–590. 18194988 10.1136/gut.2007.126680

[bib16] O'Mahony SM, Marchesi JR, Scully P, Codling C, Ceolho AM, Quigley EM et al. Early life stress alters behavior, immunity, and microbiota in rats: implications for irritable bowel syndrome and psychiatric illnesses. Biol Psychiatry 2009; 65: 263–267. 10.1016/j.biopsych.2008.06.026 18723164

[bib17] Gareau MG, Jury J, MacQueen G, Sherman PM, Perdue MH. Probiotic treatment of rat pups normalises corticosterone release and ameliorates colonic dysfunction induced by maternal separation. Gut 2007; 56: 1522–1528. 17339238 10.1136/gut.2006.117176 PMC2095679

[bib18] Callaghan BL, Richardson R. Maternal separation results in early emergence of adult-like fear and extinction learning in infant rats. Behav Neurosci 2011; 125: 20–28. 21319883 10.1037/a0022008

[bib19] Callaghan BL, Richardson R. Adverse rearing environments and persistent memories in rats: removing the brakes on infant fear memory. Transl Psychiatry 2012; 2: e138. 22781171 10.1038/tp.2012.65 PMC3410617

[bib20] Foster JA, McVey Neufeld K-A. Gut–brain axis: how the microbiome influences anxiety and depression. Trends Neurosci 2013; 36: 305–312. 23384445 10.1016/j.tins.2013.01.005

[bib21] Logan AC, Katzman M. Major depressive disorder: probiotics may be an adjuvant therapy. Med Hypotheses 2005; 64: 533–538. 15617861 10.1016/j.mehy.2004.08.019

[bib22] Sherman PM, Ossa JC, Johnson-Henry K. Unraveling mechanisms of action of probiotics. Nutr Clin Pract 2009; 24: 10–14. 19244144 10.1177/0884533608329231

[bib23] Desbonnet L, Garrett L, Clarke G, Kiely B, Cryan JF, Dinan TG. Effects of the probiotic *Bifidobacterium infantis* in the maternal separation model of depression. Neuroscience 2010; 170: 1179–1188. 20696216 10.1016/j.neuroscience.2010.08.005

[bib24] Foster LM, Tompkins TA, Dahl WJ. A comprehensive post-market review of studies on a probiotic product containing *Lactobacillus helveticus* R0052 and *Lactobacillus rhamnosus* R0011. Benef Microbes 2011; 2: 319–334. 22146691 10.3920/BM2011.0032

[bib25] Gareau MG, Wine E, Rodrigues DM, Cho JH, Whary MT, Philpott DJ et al. Bacterial infection causes stress-induced memory dysfunction in mice. Gut 2011; 60: 307–317. 20966022 10.1136/gut.2009.202515

[bib26] Fellows WD, Rasmussen KM. Comparison of methods for obtaining milk samples from well-nourished and malnourished rats. Physiol Behav 1984; 33: 761–763. 6522497 10.1016/0031-9384(84)90044-1

[bib27] Cowan CSM, Callaghan BL, Richardson R. Acute early-life stress results in premature emergence of adult-like fear retention and extinction relapse in infant rats. Behav Neurosci 2013; 127: 703–711. 24128359 10.1037/a0034118

[bib28] Josselyn SA, Frankland PW. Infantile amnesia: a neurogenic hypothesis. Learn Mem 2012; 19: 423–433. 22904373 10.1101/lm.021311.110

[bib29] Campbell BA, Campbell EH. Retention and extinction of learned fear in infant and adult rats. J Comp Psychol 1962; 55: 1–8. 10.1037/h0049182 13876002

[bib30] Kim JH, Richardson R. New findings on extinction of conditioned fear early in development: theoretical and clinical implications. Biol Psychiatry 2010; 67: 297–303. 19846065 10.1016/j.biopsych.2009.09.003

[bib31] Bravo JA, Forsythe P, Chew MV, Escaravage E, Savignac HM, Dinan TG et al. Ingestion of *Lactobacillus* strain regulates emotional behavior and central GABA receptor expression in a mouse via the vagus nerve. Proc Natl Acad Sci USA 2011; 108: 16050–16055. 21876150 10.1073/pnas.1102999108 PMC3179073

[bib32] Messaoudi M, Lalonde R, Violle N, Javelot H, Desor D, Nejdi A et al. Assessment of psychotropic-like properties of a probiotic formulation (*Lactobacillus helveticus* R0052 and *Bifidobacterium longum* R0175) in rats and human subjects. Br J Nutr 2011; 105: 755–764. 20974015 10.1017/S0007114510004319

[bib33] Walf AA, Frye CA. The use of the elevated plus maze as an assay of anxiety-related behavior in rodents. Nat Protoc 2007; 2: 322–328. 17406592 10.1038/nprot.2007.44 PMC3623971

[bib34] Lister RG. The use of a plus-maze to measure anxiety in the mouse. Psychopharmacology 1987; 92: 180–185. 3110839 10.1007/BF00177912

[bib35] Millstein RA, Holmes A. Effects of repeated maternal separation on anxiety- and depression-related phenotypes in different mouse strains. Neurosci Biobehav Rev 2007; 31: 3–17. 16950513 10.1016/j.neubiorev.2006.05.003

[bib36] Eklund MB, Arborelius L. Twice daily long maternal separations in Wistar rats decreases anxiety-like behaviour in females but does not affect males. Behav Brain Res 2006; 172: 278–285. 16780968 10.1016/j.bbr.2006.05.015

[bib37] Callaghan BL, Richardson R. Early-life stress affects extinction during critical periods of development: an analysis of the effects of maternal separation on extinction in adolescent rats. Stress 2012; 15: 671–679. 22356214 10.3109/10253890.2012.667463

[bib38] Desbonnet L, Garrett L, Clarke G, Bienenstock J, Dinan TG. The probiotic *Bifidobacteria infantis*: an assessment of potential antidepressant properties in the rat. J Psychiatr Res 2009; 43: 164–174. 10.1016/j.jpsychires.2008.03.009 18456279

[bib39] Benton D, Williams C, Brown A. Impact of consuming a milk drink containing a probiotic on mood and cognition. Eur J Clin Nutr 2006; 61: 355–361. 17151594 10.1038/sj.ejcn.1602546

[bib40] Huot RL, Gonzalez ME, Ladd CO, Thrivikraman KV, Plotsky PM. Foster litters prevent hypothalamic-pituitary-adrenal axis sensitization mediated by neonatal maternal separation. Psychoneuroendocrinology 2004; 29: 279–289. 14604606 10.1016/s0306-4530(03)00028-3

[bib41] Boccia ML, Razzoli M, Vadlamudi SP, Trumbull W, Caleffie C, Pedersen CA. Repeated long separations from pups produce depression-like behavior in rat mothers. Psychoneuroendocrinology 2007; 32: 65–71. 17118566 10.1016/j.psyneuen.2006.10.004 PMC1865504

[bib42] Maniam J, Morris MJ. Long-term postpartum anxiety and depression-like behavior in mother rats subjected to maternal separation are ameliorated by palatable high fat diet. Behav Brain Res 2010; 208: 72–79. 19896506 10.1016/j.bbr.2009.11.005

[bib43] Aguggia JP, Suárez MM, Rivarola MA. Early maternal separation: neurobehavioral consequences in mother rats. Behav Brain Res 2013; 248: 25–31. 23567892 10.1016/j.bbr.2013.03.040

[bib44] Kaffman A, Meaney MJ. Neurodevelopmental sequelae of postnatal maternal care in rodents: clinical and research implications of molecular insights. J Child Psychol Psychiatry 2007; 48: 224–244. 17355397 10.1111/j.1469-7610.2007.01730.x

[bib45] Marlin BJ, Mitre M, D/'amour JA, Chao MV, Froemke RC. Oxytocin enables maternal behaviour by balancing cortical inhibition. Nature 2015; 520: 499–504. 25874674 10.1038/nature14402 PMC4409554

[bib46] Gros DF, Antony MM, McCabe RE, Swinson RP. Frequency and severity of the symptoms of irritable bowel syndrome across the anxiety disorders and depression. J Anxiety Disord 2009; 23: 290–296. 18819774 10.1016/j.janxdis.2008.08.004

[bib47] Koloski NA, Jones M, Kalantar J, Weltman M, Zaguirre J, Talley NJ. The brain-gut pathway in functional gastrointestinal disorders is bidirectional: a 12-year prospective population-based study. Gut 2012; 61: 1284–1290. 22234979 10.1136/gutjnl-2011-300474

[bib48] Whitehead WE, Palsson O, Jones KR. Systematic review of the comorbidity of irritable bowel syndrome with other disorders: what are the causes and implications? Gastroenterology 2002; 122: 1140–1156. 11910364 10.1053/gast.2002.32392

[bib49] Lyte M, Varcoe JJ, Bailey MT. Anxiogenic effect of subclinical bacterial infection in mice in the absence of overt immune activation. Physiol Behav 1998; 65: 63–68. 9811366 10.1016/s0031-9384(98)00145-0

[bib50] Bercik P, Denou E, Collins J, Jackson WP, Lu J, Jury J et al. The intestinal microbiota affect central levels of brain-derived neurotropic factor and behavior in mice. Gastroenterology 2011; 141: 599–609. 21683077 10.1053/j.gastro.2011.04.052

[bib51] Ait-Belgnaoui A, Colon A, Braniste V, Ramalho L, Marrot A, Cartier C et al. Probiotic gut effect prevents the chronic psychological stress-induced brain activity abnormality in mice. Neurogastroenterol Motil 2014; 26: 510–520. 24372793 10.1111/nmo.12295

[bib52] Ait-Belgnaoui A, Durand H, Cartier C, Chaumaz G, Eutamene H, Ferrier L et al. Prevention of gut leakiness by a probiotic treatment leads to attenuated HPA response to an acute psychological stress in rats. Psychoneuroendocrinology 2012; 37: 1885–1895. 22541937 10.1016/j.psyneuen.2012.03.024

[bib53] Lickliter R. Premature visual stimulation accelerates intersensory functioning in bobwhite quail neonates. Dev Psychobiol 1990; 23: 015–028. 10.1002/dev.420230103 2340954

[bib54] Kenny PA, Turkewitz G. Effects of unusually early visual stimulation on the development of homing behavior in the rat pup. Dev Psychobiol 1986; 19: 57–66. 3699252 10.1002/dev.420190107

[bib55] Kagan J, Snidman N. Early childhood predictors of adult anxiety disorders. Biol Psychiatry 1999; 46: 1536–1541. 10599481 10.1016/s0006-3223(99)00137-7

[bib56] Mineka S, Zinbarg R. A contemporary learning theory perspective on the etiology of anxiety disorders: it's not what you thought it was. Am Psychol 2006; 61: 10–26. 16435973 10.1037/0003-066X.61.1.10

[bib57] Rao AV, Bested AC, Beaulne TM, Katzman MA, Iorio C, Berardi JM et al. A randomized, double-blind, placebo-controlled pilot study of a probiotic in emotional symptoms of chronic fatigue syndrome. Gut Pathog 2009; 1: 6. 19338686 10.1186/1757-4749-1-6 PMC2664325

[bib58] Tillisch K, Labus J, Kilpatrick L, Jiang Z, Stains J, Ebrat B et al. Consumption of fermented milk product with probiotic modulates brain activity. Gastroenterology 2013; 144: 1394–1401. 23474283 10.1053/j.gastro.2013.02.043 PMC3839572

[bib59] Boyle RJ, Robins-Browne RM, Tang ML. Probiotic use in clinical practice: what are the risks? Am J Clin Nutr 2006; 83: 1256–1264. 16762934 10.1093/ajcn/83.6.1256

[bib60] Barouei J, Moussavi M, Hodgson DM. Effect of maternal probiotic intervention on HPA axis, immunity and gut microbiota in a rat model of irritable bowel syndrome. PLoS One 2012; 7: e46051. 23071537 10.1371/journal.pone.0046051 PMC3469551

[bib61] Round JL, Mazmanian SK. The gut microbiota shapes intestinal immune responses during health and disease. Nat Rev Immunol 2009; 9: 313–323. 19343057 10.1038/nri2515 PMC4095778

[bib62] Mowat AM, Agace WW. Regional specialization within the intestinal immune system. Nat Rev Immunol 2014; 14: 667–685. 25234148 10.1038/nri3738

[bib63] Maes M, Yirmyia R, Noraberg J, Brene S, Hibbeln J, Perini G et al. The inflammatory & neurodegenerative (I&ND) hypothesis of depression: leads for future research and new drug developments in depression. Metab Brain Dis 2008; 24: 27–53. 19085093 10.1007/s11011-008-9118-1

[bib64] Ganguly P, Brenhouse HC. Broken or maladaptive? Altered trajectories in neuroinflammation and behavior after early life adversity. Dev Cogn Neurosci 2015; 11: 18–30. 25081071 10.1016/j.dcn.2014.07.001 PMC4476268

[bib65] Lyte M. Microbial endocrinology in the microbiome-gut-brain axis: how bacterial production and utilization of neurochemicals influence behavior. PLoS Pathog 2013; 9. 10.1371/journal.ppat.1003726 PMC3828163 24244158

[bib66] Wall R, Cryan JF, Paul Ross R, Fitzgerald GF, Dinan TG, Stanton C. Bacterial neuroactive compounds produced by psychobiotics. Adv Exp Med Biol 2014 p 817: 221–239. 24997036 10.1007/978-1-4939-0897-4_10

[bib67] Heim C, Nemeroff CB. The role of childhood trauma in the neurobiology of mood and anxiety disorders: preclinical and clinical studies. Biol Psychiatry 2001; 49: 1023–1039. 11430844 10.1016/s0006-3223(01)01157-x

[bib68] Caspi A, Sugden K, Moffitt TE, Taylor A, Craig IW, Harrington H et al. Influence of life stress on depression: moderation by a polymorphism in the 5-HTT gene. Science 2003; 301: 386–389. 12869766 10.1126/science.1083968

[bib69] O'Mahony SM, Clarke G, Borre YE, Dinan TG, Cryan JF. Serotonin, tryptophan metabolism and the brain-gut-microbiome axis. Behav Brain Res 2015; 277: 32–48. 25078296 10.1016/j.bbr.2014.07.027

[bib70] Yano JM, Yu K, Donaldson GP, Shastri GG, Ann P, Ma L et al. Indigenous bacteria from the gut microbiota regulate host serotonin biosynthesis. Cell 2015; 161: 264–276. 25860609 10.1016/j.cell.2015.02.047 PMC4393509

[bib71] Callaghan BL, Richardson R. Early emergence of adult-like fear renewal in the developing rat after chronic corticosterone treatment of the dam or the pups. Behav Neurosci 2014; 128: 594–602. 25150542 10.1037/bne0000009

