# Supplementary material for: The effects of a probiotic formulation (Lactobacillus rhamnosus and L. helveticus) on developmental trajectories of emotional learning in stressed infant rats
Source: Transl Psychiatry. 2016 May 31;6(5):e823–. doi: 10.1038/tp.2016.94 (PMC5545650; doi:10.1038/tp.2016.94)
Supplement: Supplementary Information [file tp201694x1.doc]

SupplementalMaterial:

The Effects of a Probiotic Formulation (*Lactobacillus rhamnosus and L. helveticus*) on Developmental Trajectories of Emotional Learning in Stressed Infant Rats.

Caitlin S.M. Cowan^1, *^, Bridget L. Callaghan^1,2^, & Rick Richardson^1^

^1^The University of New South Wales, School of Psychology, Sydney, 2052, Australia

^2^Columbia University, Department of Psychology, New York, 10027, USA

^3^Department of Psychiatry, The University of Melbourne, Level 1 North, Main Block Royal

Melbourne Hospital, Victoria, 3050, Australia.

**Running Title:** Probiotics & Fear Regulation in Early-Life Stress

**Corresponding Author:** Caitlin S.M. Cowan

***Address:*** School of Psychology, Level 10 Mathews Building, The University of New South Wales, NSW, Australia, 2052
***Telephone:*** +61 409227281

***Fax:*** +61 293853641
***E-mail:***[c.cowan@unsw.edu.au](mailto:c.cowan@unsw.edu.au)

Supplemental Methods

*Stomach Milk Collection.* Stomach contents were extracted from pups on P7. After maternal separation (MS), pups were reunited with the dam for 1-2 h to allow nursing to occur. After this period, pups were removed from the home cage and heavily anaesthetized by intraperitoneal injection of sodium pentobarbital (100 mg/kg). Abdominal incisions were made and the whole stomach was excised and rinsed in sterile saline. A small incision was made in the stomach wall to allow the curdled stomach milk to be collected. All samples werestored at −20°C until analysis.

*DNA Extraction.* DNA was extracted from stomach milk samples (400 mg ± 1 mg) using a milk bacterial DNA isolation kit (NorgenBiotek Corporation, Thorold, ON, Canada). Curdled milk was first diluted and homogenized in 4 ml distilled water. For one of the samples collected from a vehicle-exposed MS pup, the original specimen was divided into two 400 mg samples and one of these was diluted in probiotic solution (10^9^ CFU/ml in distilled water; i.e., the same concentration as the maternal drinking water) to create a positive control. One milliliter of the diluted milk solution was used for the extraction, which was conducted following the manufacturer instructions.Extracted DNA was stored at 4°C until qPCR. DNA extraction and subsequent qPCR was conducted by an experimenter blinded to the experimental condition of each sample.

*qPCR.* Quantitative real-time polymerase chain reaction (qPCR) was conducted on the Applied BiosystemsStepOnePlus Real-Time PCR System. Specific forward and reverse primers for *L. rhamnosus* strain R0011 specified by previous research (1) were obtained from Thermo Fisher Scientific (forward primer sequence: 5′ TCAGTAGACACCTACCGG; reverse primer sequence: 5′ GTTGTAAAAGCTCTGGGACGCG). The qPCR mixture (total volume: 20µl) consisted of 5µl template DNA, 10µL PowerUp SYBR MasterMix (Thermo Fisher Scientific), and 0.5µM of each primer in DEPC-treated water. qPCR conditions included 40 cycles of denaturation (95ºC, 15s) and annealing (61ºC, 60s). Following this process, melting curves were generated to verify reaction specificity, resulting in a single peak for each amplified well at approximately 82.6ºC (Supplemental Figure S1).


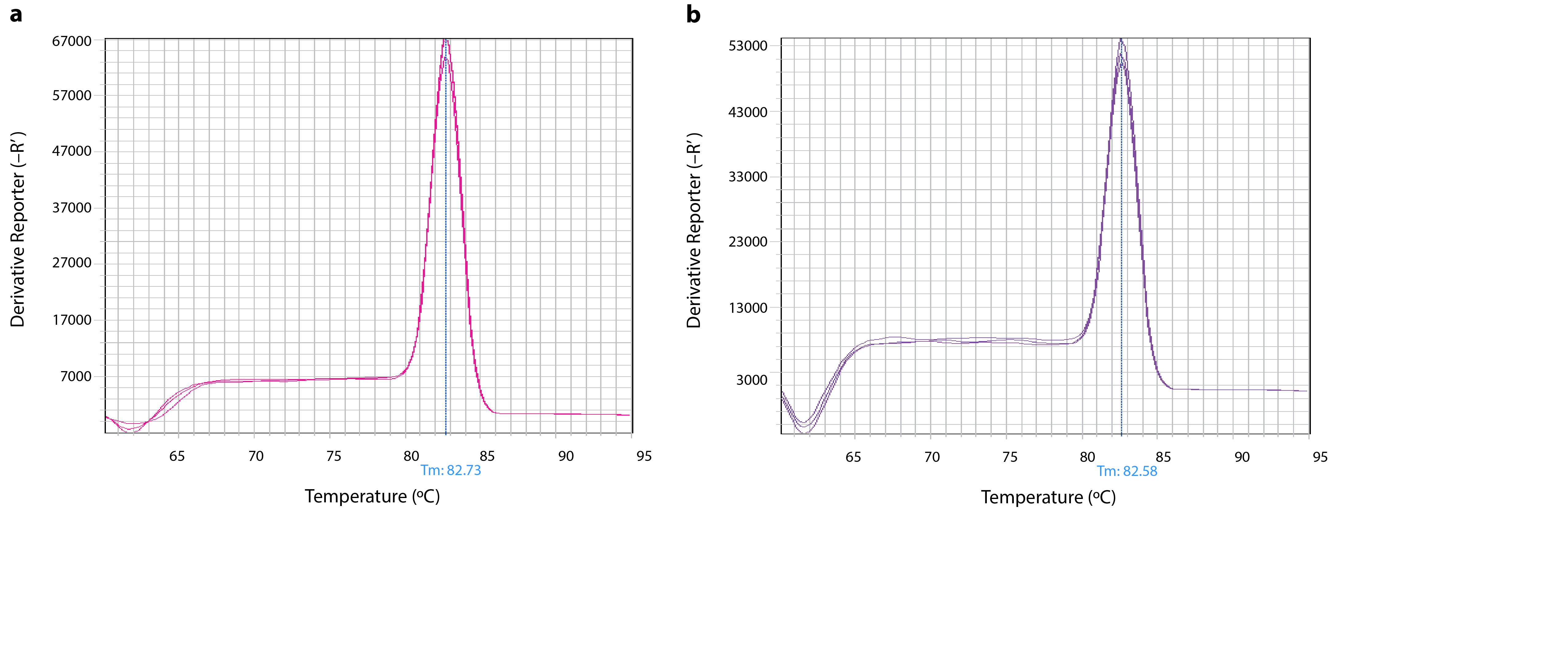


**Supplemental Figure S1:qPCR melting curves.**Melting curves for the qPCR analysis of *L. rhamnosus* strain R0011 from (a) the positive control and (b) a representative stomach milk sample from a probiotic-exposed MS animal.

Supplemental Results

*Baseline Freezing – Experiment 1.* Freezing was measured for 1 minute prior to the CS onset at test (Supplemental Table S1). The effect of treatment and the Treatment x Test Interval interaction were non-significant (*F*s < 1) but there was a significant effect of test interval (*F*_1,46_ = 19.73, *p*< .001). That is, rats exhibited higher levels of baseline freezing in the testing chamber (which was the same as the training chamber) 1 day after training compared to 7 days after training. However, probiotic treatment did not affect baseline freezing.

*Baseline Freezing – Experiment 2: Extinction.* Freezing was measured for 2 minutes prior to onset of the first CS presentation (Supplemental Table S1). The effects of context and the Treatment x Test Context interaction were non-significant (*F*s < 1). However, there was a significant effect of treatment (*F*_1,47_ = 4.51, *p* = .04) such that probiotic-exposed animals exhibited higher levels of baseline freezing compared to vehicle-exposed animals. Although this might indicate that probiotic treatment leads to increases in generalized fear in the MS infants, it is unclear whether this result is robust; this was the only case where differences in baseline freezing were observed between treatment groups and the results of Experiment 3a suggest that the probiotic-exposed infants did not exhibit differences in diffuse fear or anxiety on the elevated-plus maze.

*Baseline Freezing – Experiment 2: Test.* As in Experiment 1, baseline freezing was measured for 1 minute prior to CS onset (Supplemental Table S1). The effect of treatment and the Treatment x Test Context interaction were non-significant (*F*s < 1) but there was a significant effect of test context(*F*_1,47_ = 4.16, *p* = .047). This effect was due to the MS infant rats tested in the training context exhibiting higher levels of freezing compared to those tested in the extinction context. However, baseline freezing was not affected by probiotic treatment.

**Supplemental Table S1.** Percentage Baseline Freezing for Vehicle and Probiotic Treatment Groups in Experiments 1 and 2

|  | **Vehicle** | | **Probiotic** | |
| --- | --- | --- | --- | --- |
| **Experiment & Group** | ***N*** | ***M* (± *SEM*)** | ***n*** | ***M* (± *SEM*)** |
| Expt 1: 1 Day | 14 | 29.83 (± 4.75) | 13 | 25.77 (± 5.95) |
| Expt 1: 7 Days | 13 | 9.62 (± 3.88) | 10 | 5.50 (± 2.88) |
| Expt 2: Same Context - Extinction | 11 | 11.25 (± 2.87) | 15 | 25.41 (± 5.77) |
| Expt 2: Different Context - Extinction | 11 | 17.62 (± 6.56) | 14 | 26.38 (± 5.67) |
| Expt 2: Same Context - Test | 11 | 3.73 (± 1.08) | 15 | 3.71 (± 2.57) |
| Expt 2: Different Context - Test | 11 | 9.29 (± 4.38) | 14 | 11.79 (± 4.37) |

*Dam Fluid Intake.* Fluid consumption of 26 vehicle-treated dams and 21 probiotic-treated dams was monitored daily by measuring water bottle weights. There were 12 missing observations out of 611 (i.e., 2.0% of the total) due to water bottle leakage or missed data collection. These missing data points were estimated by taking the dam’s average consumption across the remainder of the days.

**
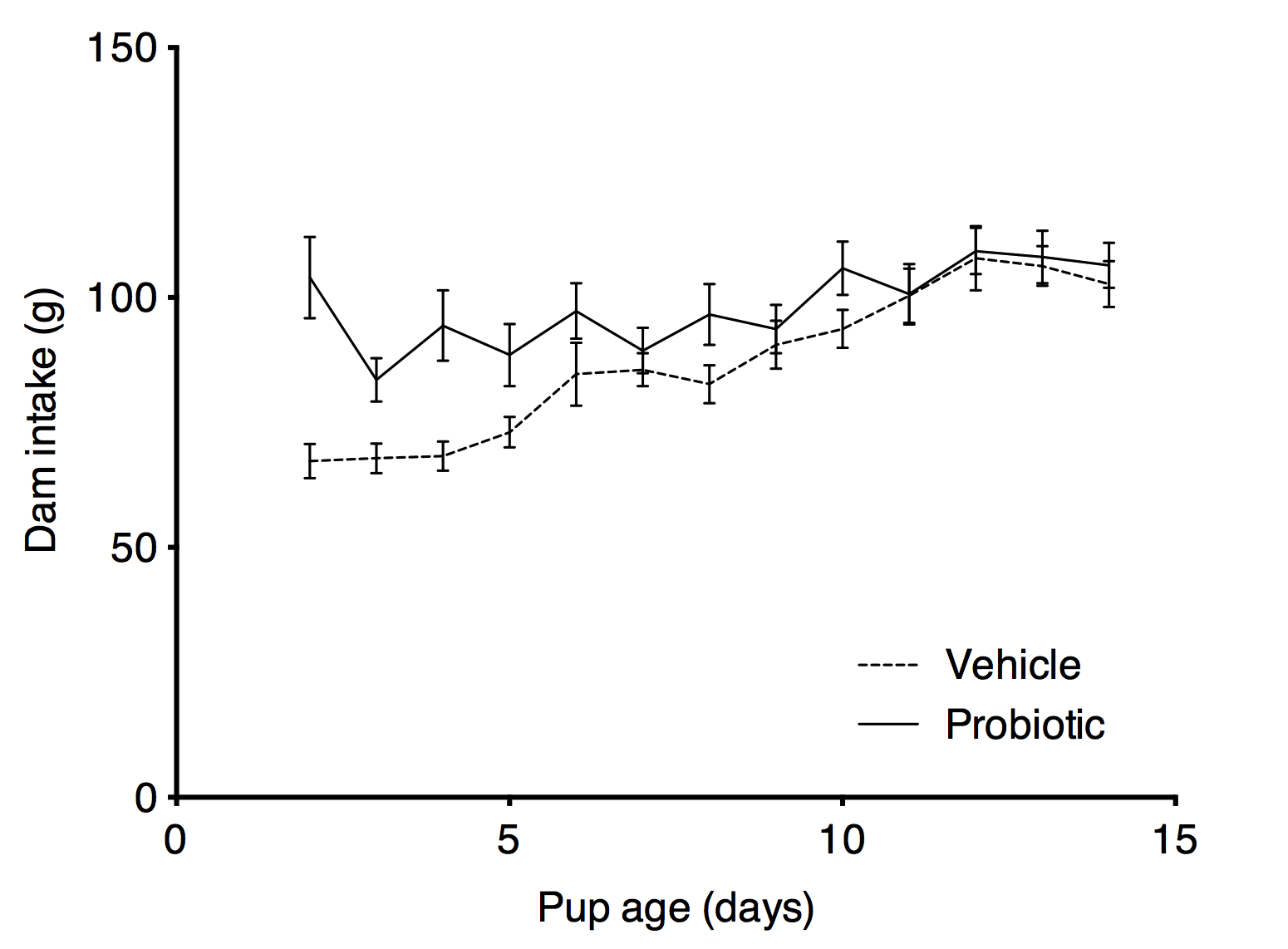
**

**Supplemental Figure S2: Dam fluid intake.** Mean (± *SEM*) consumption by dams provided with vehicle (distilled water) or probiotic during treatment (from postnatal days 2-14).

Intake increased with pups’ age for both treatment types (Figure S1). However, probiotic-treated dams consumed more than vehicle-treated dams, particularly in the first few days of treatment. This description was confirmed by the statistical analysis, with significant main effects of day (*F*_12,540_ = 15.74, *p*< .001) and treatment type (*F*_1,45_ = 6.27, *p* = .02), as well as a significant Day x Treatment Type interaction (*F*_12,540_ = 4.16, *p*< .001). Follow-up analysis was conducted on dams’ average intake across the initial (P2-4), middle (P7-9), and final (P12-14) periods of treatment, revealing that the difference in intake was limited to the start of treatment. In the initial period, dams in the probiotic-treated group consumed more than vehicle-treated dams (*t*_45_ = 4.71, *p*< .001) but the difference between groups was non-significant in both the middle and final periods of treatment (largest *t*_45_ = 1.34, *p* = .19).

*Animal Weights.* Pups from 17 vehicle-exposed litters and 15 probiotic-exposed litters were weighed daily as a litter and the average taken to obtain pup weights. Nine vehicle-treated dams and 7 probiotic-treated dams were also weighed daily. For the dams, there were 4 missing data points out of 208 (i.e., 1.9% of the total) that were estimated by taking the dam’s average weight across the remainder of the days.

Pups gained weight across development, whereas dams lost weight over the same period (Supplemental Figure S2). The statistical analyses confirmed significant effects of pup age (pups: *F*_12,360_ = 2064.91, *p*< .001, dams: *F*_12,168_ = 5.69, *p*< .001,), but the effects of treatment type and the Pup Age x Treatment Type interactions were non-significant (pups: largest *F*_1,30_ = 1.03, *p* = .32, dams: largest *F*_12,168_ = 1.03, *p* = .41).


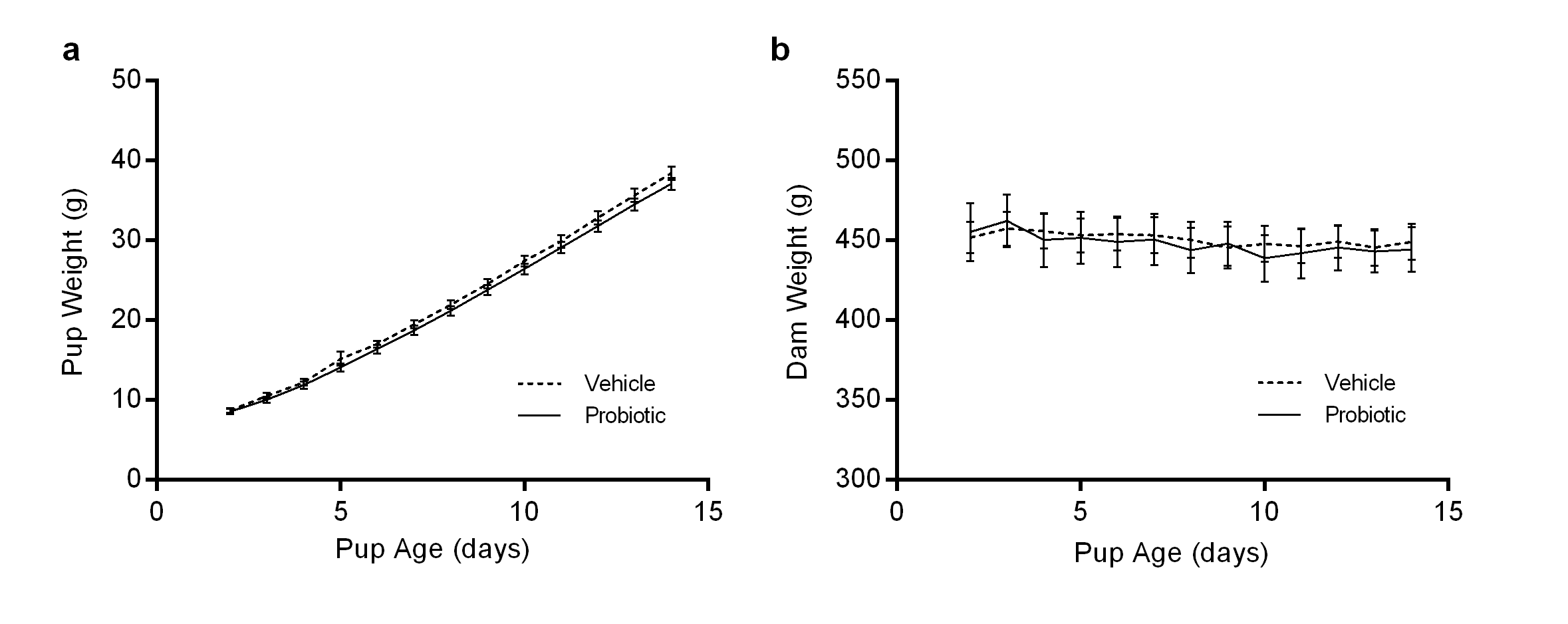


**Supplemental Figure S3: Animal body weights.** Mean (± *SEM*)body weight of (a) pups and (b) dams as a function of pups’ age.

*Experiment 3.* Additional measures of anxiety-like behavior on the elevated-plus maze are presented in Supplemental Figure S4. No differences between groups were observed – see main manuscript for statistical analysis.

**Supplemental Figure S4: Additional elevated-plus maze (EPM) measures.** Mean (± *SEM*) number of total arm entries and latency to enter open arms for (a-b) pups and (c-d) dams on the EPM.

**References**

1. Gareau MG, Jury J, MacQueen G, Sherman PM, Perdue MH. Probiotic treatment of rat pups normalises corticosterone release and ameliorates colonic dysfunction induced by maternal separation*. G*ut 2007**; 56**: 1522-1528.
